# Case Report: Chronic expanding axillary hematoma mimicking locoregional recurrence 15 years after breast cancer surgery

**DOI:** 10.3389/fonc.2026.1934091

**Published:** 2026-09-17

**Authors:** Sen Li, Yingfang Shi, Zhichun Wang

**Affiliations:** Breast Surgery, Jiujiang City Key Laboratory of Cell Therapy, JiuJiang No.1 People’s Hospital, Jiujiang, Jiangxi, China

**Keywords:** axillary mass, breast cancer, case report, chronic expanding hematoma, locoregional recurrence, radiation-associated angiosarcoma, radiotherapy

## Abstract

Chronic expanding hematoma (CEH) is a rare condition characterized by progressive enlargement of a hematoma long after an initial hemorrhagic event and may mimic malignancy. We report a 61-year-old woman who developed a progressively enlarging left axillary mass 15 years after modified radical mastectomy followed by adjuvant chemotherapy and radiotherapy for breast cancer. The lesion became ulcerated and spontaneously bled, raising concern for locoregional recurrence. Ultrasonography demonstrated heterogeneous mixed cystic-solid lesions, while systemic evaluation revealed no evidence of distant metastatic disease. Ultrasound-guided core needle biopsy showed hemorrhage, fibrin deposition, and fibrous tissue without malignant cells but was considered inconclusive because of potential sampling limitations. Surgical excision and extensive debridement were therefore performed. The 7 × 5 × 2 cm excised specimen was extensively sampled in 20 tissue blocks and demonstrated organized hematoma, fibrosis, hemosiderin-laden macrophages, and chronic inflammatory changes, without atypical endothelial proliferation or malignant tumor cells. CEH was definitively diagnosed. Following staged wound management with vacuum sealing drainage, the axillary defect was successfully reconstructed using a pedicled scapular artery perforator propeller flap. This case emphasizes the importance of considering CEH, recurrent carcinoma, and angiosarcoma in the differential diagnosis of delayed hemorrhagic masses after breast cancer treatment and highlights the importance of adequate histopathological sampling for definitive diagnosis.

## Background

Chronic expanding hematoma (CEH) is an uncommon clinicopathological entity characterized by the progressive enlargement of a hematoma over months or even years after the initial hemorrhagic event (1–4). First described by Reid et al. in 1980, CEH is histologically characterized by a fibrous capsule surrounding organized hematoma with repeated microhemorrhage from fragile neovascularized capillaries, resulting in continuous expansion of the lesion (1, 2). CEH has been associated with previous trauma, surgery, and radiotherapy and may clinically and radiologically mimic malignant soft-tissue tumors because of its progressive growth and heterogeneous imaging features (3–8). Although CEH has been reported in the thoracic cavity, chest wall, extremities, retroperitoneum, and trunk, axillary involvement remains exceedingly uncommon, particularly following breast cancer treatment (4, 7–10).

The diagnosis of CEH remains challenging because patients with a history of breast cancer who develop a progressively enlarging axillary mass are generally suspected of having locoregional recurrence or metastatic lymphadenopathy. Imaging findings are frequently nonspecific, and histopathological examination is often required to establish the definitive diagnosis (5, 6, 11–13). Consequently, delayed postoperative CEH may lead to unnecessary anxiety, repeated invasive investigations, or inappropriate oncologic treatment if not recognized promptly (11–13).

Herein, we report a patient with chronic expanding axillary hematoma that developed 15 years after modified radical mastectomy and adjuvant radiotherapy for breast cancer, clinically and radiologically mimicking locoregional recurrence. To the best of our knowledge, this represents one of the very few reported cases of delayed axillary CEH following breast cancer treatment (14–16). In addition, unlike previously reported cases, our patient underwent staged surgical management with vacuum sealing drainage followed by pedicled scapular artery perforator propeller flap reconstruction, achieving a favorable early functional and reconstructive outcome.

## Case presentation

A 61-year-old woman presented to our institution in August 2025 with a one-year history of a progressively enlarging left axillary mass accompanied by spontaneous bleeding for 2 hours before admission. In 2010, she underwent left modified radical mastectomy for breast cancer, followed by adjuvant chemotherapy and radiotherapy. Because the treatment had been performed approximately 15 years earlier, the complete original pathological and treatment records were no longer available for review. Therefore, the exact histological subtype, pathological TNM stage, ER/PR/HER2 status, specific chemotherapy regimen, and radiotherapy field and dose could not be reliably retrieved. Subsequent routine surveillance showed no documented locoregional recurrence or distant metastasis before the current presentation.

Physical examination revealed a firm, poorly mobile mass measuring approximately 3.0 × 3.0 cm in the left axilla with overlying skin ulceration, active bleeding, and surrounding inflammatory changes (**Figures 1A,B**). No palpable supraclavicular lymphadenopathy was identified. Routine laboratory investigations were unremarkable except for mildly elevated inflammatory markers.

**Figure 1:**
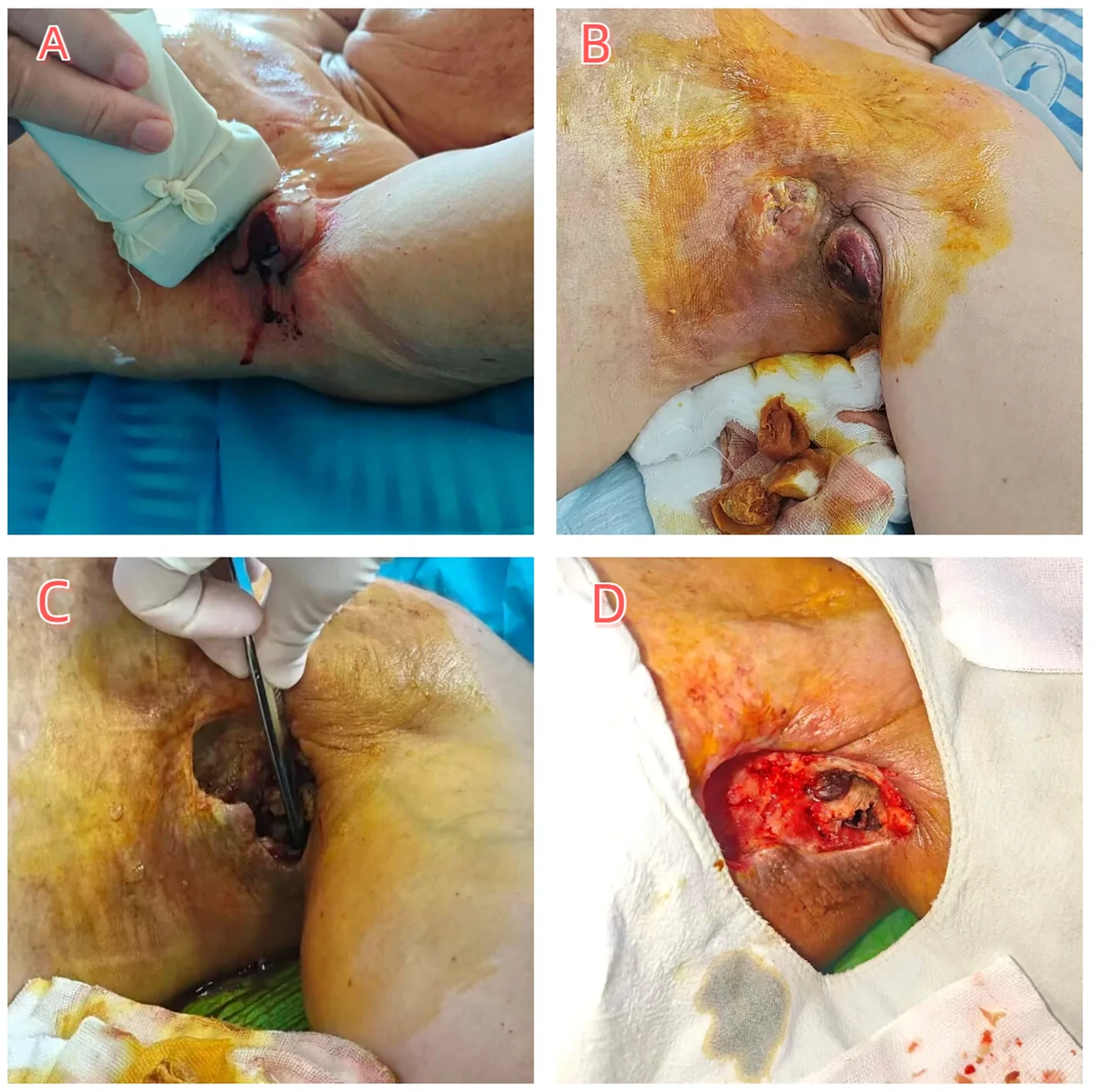
Clinical presentation and staged wound management. **(A)** Initial clinical appearance of the left axillary lesion with active spontaneous bleeding. **(B)** Close-up view showing ulceration, necrotic change, and surrounding inflammatory changes. **(C)** Appearance of the ulcerated axillary lesion during local assessment and debridement. **(D)** Wound appearance after extensive debridement, demonstrating the residual axillary soft-tissue defect before definitive reconstruction.

Ultrasonography demonstrated multiple confluent heterogeneous mixed cystic-solid lesions in the left axilla, with the largest measuring approximately 49.1 × 36.4 mm (**Figure 2A**). Based on the patient’s history of breast cancer and the imaging findings, locoregional recurrence with metastatic axillary lymphadenopathy was initially suspected. Given this concern, systemic evaluation for possible recurrent disease was performed, including chest computed tomography (CT), abdominal ultrasonography, and serum tumor marker testing. Chest CT and abdominal ultrasonography revealed no evidence of distant metastatic disease, and the serum tumor markers were within the reference ranges.

**Figure 2:**
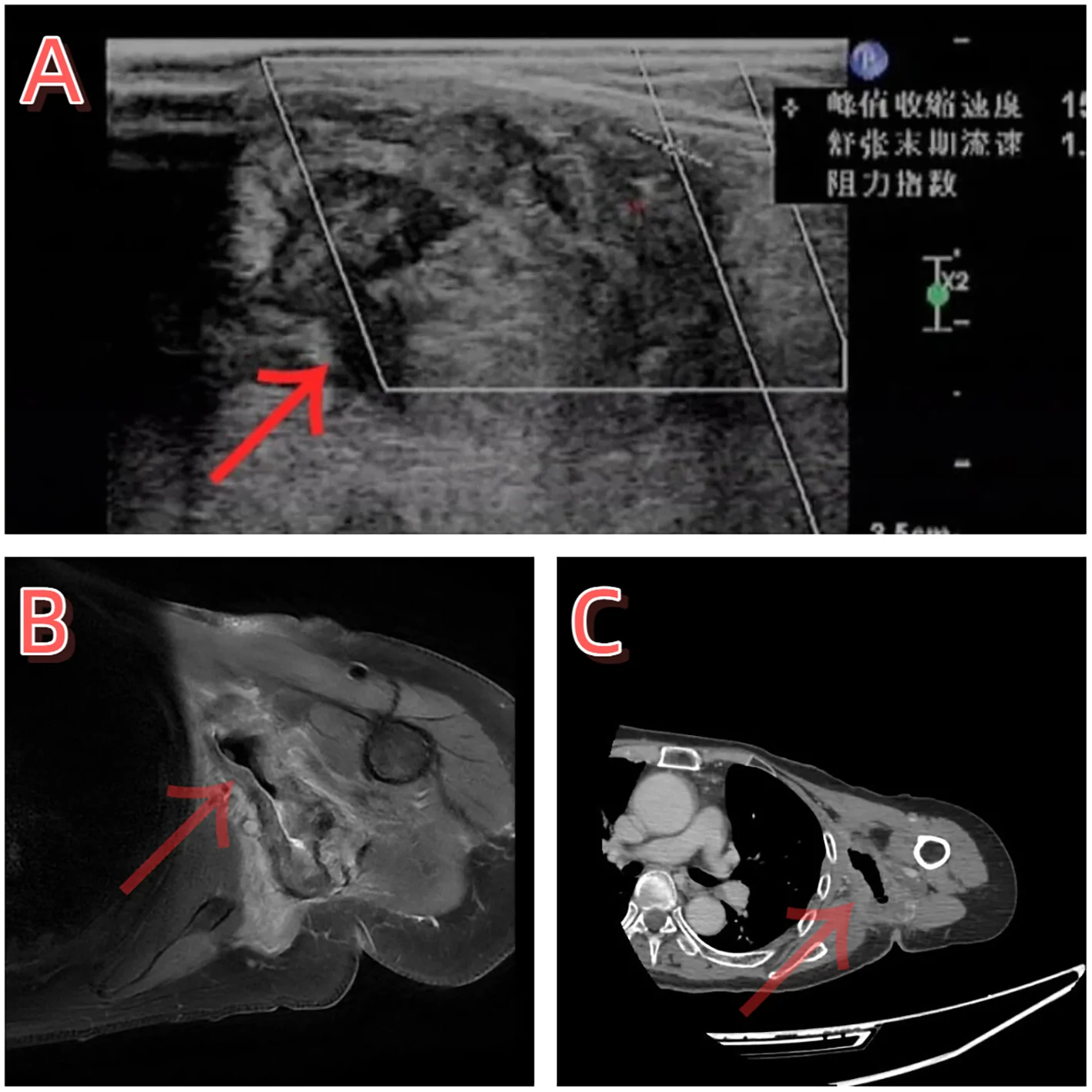
Diagnostic imaging findings. **(A)** Ultrasonography demonstrating multiple confluent heterogeneous mixed cystic-solid lesions in the left axilla, with the largest measuring approximately 49.1 × 36.4 mm; the red arrow indicates the lesion. **(B)** Contrast-enhanced magnetic resonance imaging showing heterogeneous thickening and abnormal signal/enhancement in the left axillary soft tissues; the red arrow indicates the involved region. **(C)** Computed tomography angiography demonstrating the left axillary soft-tissue abnormality without active arterial bleeding or major vascular abnormality; the red arrow indicates the affected axillary region.

To establish a tissue diagnosis, ultrasound-guided core needle biopsy was performed on August 8, 2025. Histopathological examination demonstrated extensive hemorrhage, fibrin deposition, and a small amount of fibrous connective tissue without identifiable malignant cells (**Figures 3A,B**). Because the lesion was heterogeneous and the biopsy provided limited tissue, this result was considered nondiagnostic rather than definitive; recurrent breast carcinoma or angiosarcoma could not be reliably excluded on the basis of the core biopsy alone (17).

**Figure 3:**
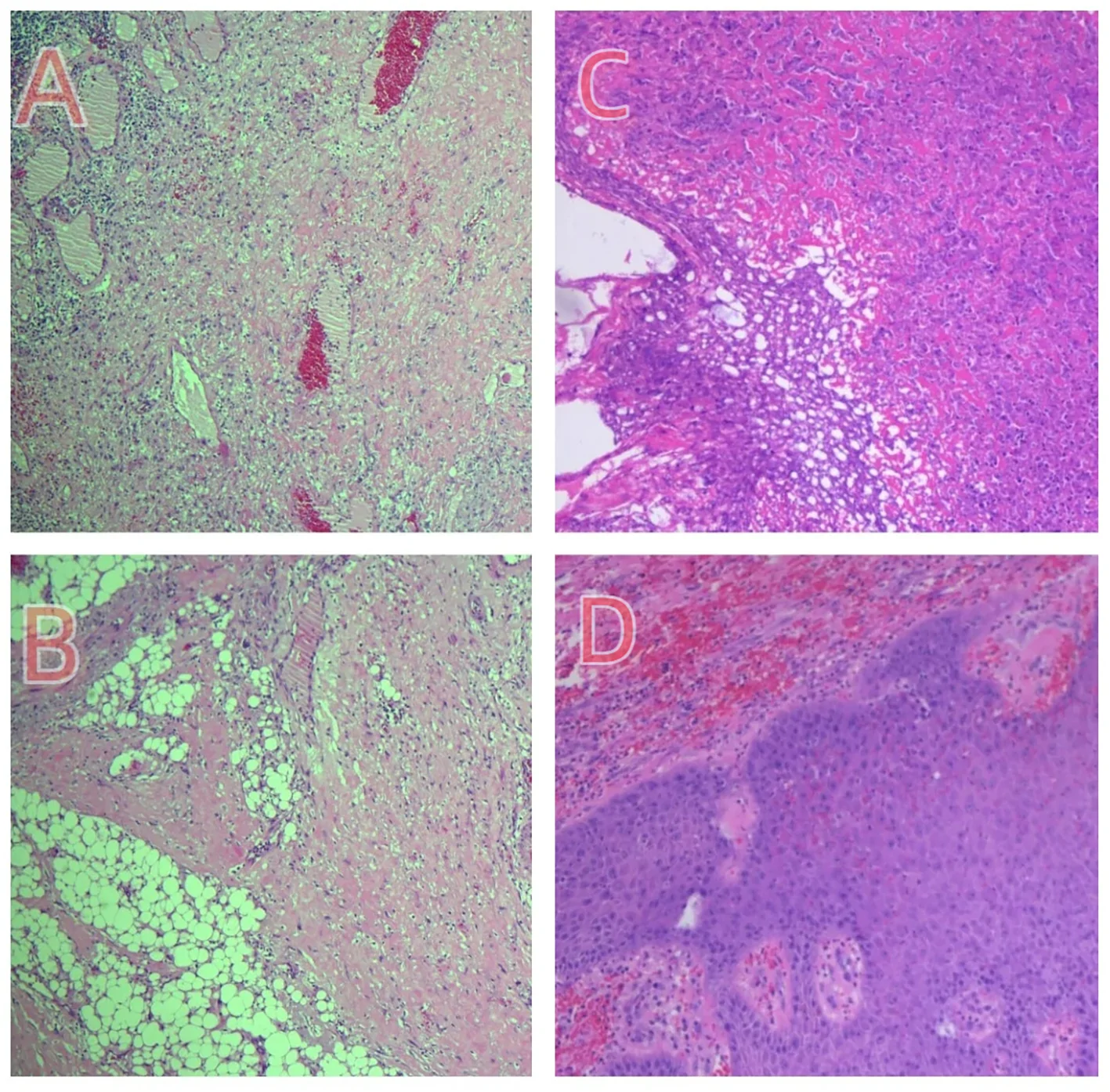
Histopathological findings of the left axillary lesion. **(A, B)** Ultrasound-guided core needle biopsy specimens showing extensive hemorrhage, fibrin deposition, inflammatory changes, and fibrous connective tissue without identifiable malignant cells (hematoxylin and eosin staining; original magnification, ×100). **(C, D)** Histopathological examination of the extensively sampled excised lesion showing an organized hematoma with extensive hemorrhage, fibrosis, hemosiderin-laden macrophages, and chronic inflammatory changes, without atypical endothelial proliferation or malignant tumor cells (hematoxylin and eosin staining; original magnification, ×100).

Following the biopsy, the patient developed persistent wound exudation and fever. Laboratory investigations demonstrated leukocytosis with neutrophilia, consistent with secondary wound infection. Cefuroxime sodium (1.5 g intravenously every 8 hours) was initially administered. Because of persistent local inflammatory manifestations, antimicrobial therapy was subsequently changed to piperacillin sodium/tazobactam sodium (4.5 g intravenously every 8 hours). After antimicrobial therapy and meticulous wound care, the patient’s body temperature returned to normal, inflammatory markers gradually improved, and wound exudation markedly decreased. However, because malignancy could not be confidently excluded and the axillary mass persisted, definitive surgical treatment was recommended.

On August 19, 2025, the patient underwent excision of the left axillary lesion with extensive debridement under general anesthesia. Intraoperatively, large amounts of organized hematoma, necrotic tissue, and fibrotic debris were identified within the lesion cavity. The excised specimen measured 7 × 5 × 2 cm and was extensively sampled in 20 tissue blocks, including the fibrous capsule, ulcerated and hemorrhagic areas, solid areas, and peripheral tissue. Histopathological examination demonstrated an organized hematoma with extensive hemorrhage, fibrin deposition, fibrosis, hemosiderin-laden macrophages, and chronic inflammatory changes. No atypical endothelial proliferation or malignant epithelial/tumor cells were identified, and the surgical margins were negative for malignancy. Because extensive histological evaluation revealed no morphologically suspicious epithelial or vascular proliferation requiring further immunophenotypic characterization, additional immunohistochemical staining was not considered necessary. These findings established the definitive diagnosis of chronic expanding hematoma (**Figures 3C,D**).

Because extensive soft-tissue loss remained after debridement, a second-stage procedure consisting of repeated debridement and vacuum sealing drainage (VSD) was performed on August 22, 2025. Continuous negative-pressure wound therapy and regular dressing changes promoted effective drainage and healthy granulation tissue formation, while repeated bacterial cultures remained negative (**Figures 1C, D**).

After the immediate diagnostic and wound-management issues had been addressed, contrast-enhanced magnetic resonance imaging (MRI) and computed tomography angiography (CTA) were subsequently performed to characterize the residual axillary soft tissues and facilitate definitive reconstructive planning. MRI demonstrated heterogeneous thickening of the left axillary soft tissues with multiple nodular and linear enhancing lesions (**Figure 2B**). CTA revealed no active arterial bleeding or major vascular abnormalities and provided anatomical information regarding the regional vasculature relevant to pedicled flap reconstruction (**Figure 2C**).

After satisfactory infection control and wound bed preparation, definitive reconstruction was performed on October 16, 2025 using a pedicled scapular artery perforator propeller flap measuring approximately 7.5 × 6.0 cm. The flap demonstrated excellent perfusion intraoperatively and remained completely viable throughout the postoperative period (**Figures 4A–D**).

**Figure 4:**
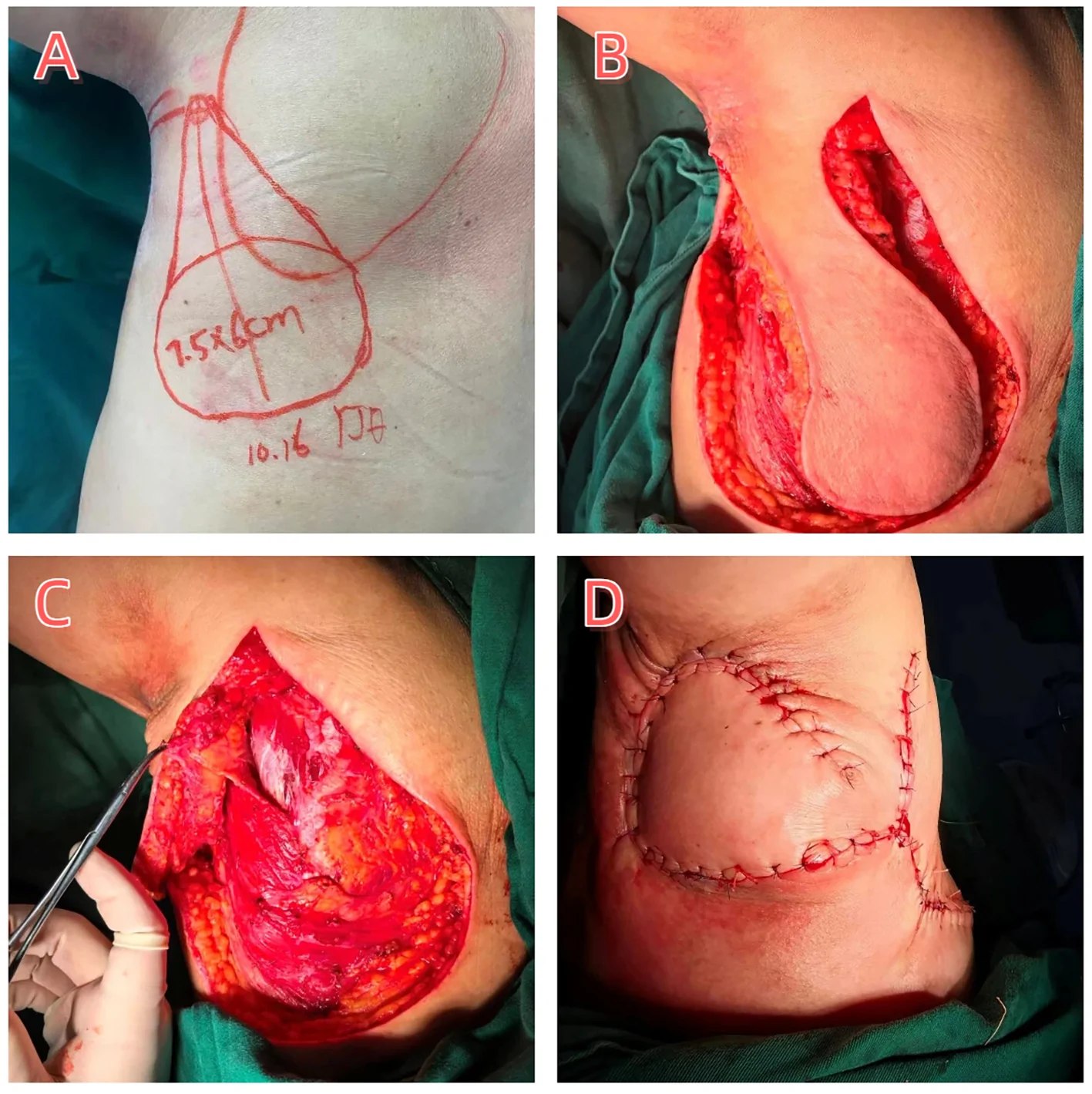
Surgical reconstruction using a pedicled scapular artery perforator propeller flap. **(A)** Preoperative design of the pedicled scapular artery perforator propeller flap. **(B)** Flap elevation and rotation into the axillary defect after wound-bed preparation. **(C)** Intraoperative view of the prepared axillary defect and flap inset. **(D)** Immediate postoperative appearance after flap transposition and wound closure, demonstrating complete defect coverage.

The postoperative course was uneventful. No flap ischemia, necrosis, wound dehiscence, or recurrent infection occurred. At the latest follow-up in May 2026, approximately seven months after definitive reconstruction, the surgical wound had healed completely, the flap remained viable, and functional and cosmetic outcomes were satisfactory. No clinical evidence of recurrent hematoma, local infection, or malignancy was observed. Continued clinical and imaging surveillance was planned.

## Clinical timeline

2010: Left modified radical mastectomy for breast cancer followed by adjuvant chemotherapy and radiotherapy. Approximately 2024-2025: Progressive enlargement of a left axillary mass. August 2025: Presentation with ulceration and spontaneous bleeding; ultrasonography raised suspicion for locoregional recurrence, while chest CT, abdominal ultrasonography, and serum tumor markers showed no evidence of distant metastatic disease. August 8, 2025: Ultrasound-guided core needle biopsy was nondiagnostic because of limited sampling. August 19, 2025: Excision and extensive debridement; a 7 × 5 × 2 cm specimen was sampled in 20 tissue blocks and established the diagnosis of CEH with no morphological evidence of carcinoma or angiosarcoma. August 22, 2025: Repeat debridement and VSD. Subsequently: MRI and CTA for residual soft-tissue assessment and reconstructive planning. October 16, 2025: Definitive reconstruction with a pedicled scapular artery perforator propeller flap. May 2026: Complete wound healing with satisfactory functional and cosmetic outcomes and no clinical evidence of recurrent hematoma, infection, or malignancy.

## Patient perspective

At the latest follow-up, the patient reported satisfactory recovery after reconstruction, with no major wound-related discomfort or functional limitation affecting daily activities. She was satisfied with the wound healing and reconstructive outcome.

## Discussion

CEH is an uncommon late complication that should be considered in patients presenting with a progressively enlarging mass after previous surgery or radiotherapy (1, 3, 4, 7–10). In patients with a history of breast cancer, however, a delayed axillary mass raises immediate concern for locoregional recurrence or metastatic lymphadenopathy. In the present case, the patient presented 15 years after modified radical mastectomy and adjuvant radiotherapy with a progressively enlarging axillary mass accompanied by spontaneous bleeding. The prolonged latency, previous oncological history, ulceration, bleeding, and heterogeneous imaging findings raised concern not only for recurrent breast carcinoma but also for a hemorrhagic soft-tissue malignancy, including radiation-associated angiosarcoma. The core needle biopsy did not identify malignant cells, but this result was considered nondiagnostic because of limited sampling in a heterogeneous lesion. Complete surgical excision with extensive pathological evaluation was therefore required to establish the definitive diagnosis.

CEH was first described by Reid et al. in 1980 as a hematoma that persists and gradually enlarges for more than one month after the initial hemorrhagic event (1). Unlike ordinary hematomas, CEH continues to expand because repeated microhemorrhage from fragile neovascularized capillaries within a fibrous capsule perpetuates a cycle of bleeding, chronic inflammation, fibrosis, and capsule formation. Previous surgery and radiotherapy have been reported as potential predisposing factors for CEH (3–10, 14–16). Proposed mechanisms include chronic vascular injury, tissue fibrosis, impaired lymphatic drainage, and persistent inflammation. In the present case, development of CEH after a prolonged latency in a previously operated and irradiated anatomical region raises the possibility of an association with prior local treatment. However, a direct causal relationship between radiotherapy and CEH cannot be established from this single case, particularly because detailed information regarding the original radiotherapy field and dose was unavailable. Other mechanisms contributing to recurrent local hemorrhage cannot be excluded.

One of the greatest clinical challenges is distinguishing CEH from recurrent breast carcinoma and angiosarcoma. Radiation-associated angiosarcoma is an important differential diagnosis in a previously irradiated breast cancer survivor presenting with a delayed enlarging, ulcerated, or hemorrhagic lesion. In addition, angiosarcoma arising in association with CEH has been reported (17). Extensive hemorrhage, necrosis, and fibrosis can make both CEH and hemorrhagic malignancies heterogeneous, creating a substantial risk of sampling error with core needle biopsy. Therefore, the absence of malignant cells in a limited biopsy specimen should not be regarded as sufficient to exclude malignancy when clinical suspicion persists. In our patient, the excised specimen measured 7 × 5 × 2 cm and was extensively sampled in 20 tissue blocks, including the fibrous capsule, ulcerated and hemorrhagic areas, solid areas, and peripheral tissue. Histological examination showed organized hematoma, extensive hemorrhage, fibrin deposition, fibrosis, hemosiderin-laden macrophages, and chronic inflammatory changes, with no atypical endothelial proliferation or malignant epithelial/tumor cells; the surgical margins were negative for malignancy. Because no morphologically suspicious vascular or epithelial proliferation was identified despite extensive sampling, additional immunohistochemical characterization was not considered necessary. These findings provided no morphological evidence of either recurrent breast carcinoma or angiosarcoma. Nevertheless, given the previous radiotherapy, prolonged latency, and reported association between CEH and angiosarcoma, continued clinical and imaging surveillance remains warranted.

Imaging plays complementary but distinct roles during evaluation and treatment planning. Ultrasonography was used initially because the lesion was superficial and readily accessible, and tissue diagnosis was prioritized because imaging appearances of CEH and malignant lesions may overlap. Systemic evaluation with chest CT, abdominal ultrasonography, and serum tumor markers showed no evidence of distant metastatic disease. MRI and CTA were obtained subsequently, after the immediate diagnostic and wound-management issues had been addressed, when the clinical objective had shifted toward characterization of residual soft-tissue changes and reconstructive planning. MRI delineated the residual axillary soft tissues, whereas CTA defined regional vascular anatomy relevant to flap reconstruction. Although pre-biopsy cross-sectional imaging could have provided additional anatomical information, it would not have replaced histopathological confirmation because CEH, recurrent carcinoma, soft-tissue sarcoma, and chronic inflammatory lesions may have overlapping imaging appearances (5, 6, 11–13). Complete surgical excision is currently considered the treatment of choice for CEH because conservative treatment or incomplete evacuation may result in persistent bleeding or recurrence (7, 8, 18). In our patient, extensive debridement created a large axillary soft-tissue defect that precluded primary closure. VSD was used to control local wound problems and optimize the wound bed (19), followed by definitive reconstruction using a pedicled scapular artery perforator propeller flap (20–22). This staged strategy provided effective soft-tissue coverage and a favorable early functional and reconstructive outcome.

This case provides several important clinical implications. First, CEH should be included in the differential diagnosis of delayed axillary masses developing years after breast cancer surgery and radiotherapy, even when imaging strongly suggests locoregional recurrence. Second, recurrent breast carcinoma, radiation-associated angiosarcoma, and CEH-associated angiosarcoma should also be considered in an enlarging hemorrhagic lesion in a previously treated field. Third, a negative core needle biopsy may be insufficient in a heterogeneous lesion; when clinical suspicion persists, adequate excision and extensive histopathological sampling are essential. Finally, previous surgery and radiotherapy may represent potential predisposing factors for CEH, but causality cannot be established from this single case. An additional limitation is the unavailability of the complete records from the patient’s original breast cancer treatment in 2010. Although left modified radical mastectomy followed by adjuvant chemotherapy and radiotherapy could be confirmed, the original histological subtype, pathological TNM stage, receptor status, specific chemotherapy regimen, and radiotherapy field and dose could not be reliably retrieved because of the prolonged interval. This limits detailed assessment of the relationship between the original breast cancer treatment and the subsequent axillary lesion.

## Conclusion

This case highlights that chronic expanding hematoma should be considered in the differential diagnosis of delayed axillary masses occurring years after breast cancer surgery and radiotherapy. Recurrent breast carcinoma and angiosarcoma, including radiation-associated angiosarcoma, must also be considered in a progressively enlarging hemorrhagic lesion. Because imaging findings and limited biopsy specimens may be nondiagnostic, definitive diagnosis may require complete excision with adequate histopathological sampling. Complete surgical excision followed by individualized reconstructive management can provide effective local control and favorable early postoperative outcomes. Continued clinical and imaging surveillance remains warranted, particularly in patients with a history of radiotherapy.
